# “ProTeggiMI”: Adherence to HIV Vertical Transmission Prevention Pathway in Remote Communities in Mozambique

**DOI:** 10.1093/ofid/ofaf647

**Published:** 2025-10-15

**Authors:** Carlo Cerini, Paola Magro, Aldorada da Gloria Julio Andrè, Benedetta Rossi, Dorcia Mandlate, Olivia Chambule Moçambique, Federica Boniotti, Natalia Gregori, Francesca Pennati, Verena Crosato, Pietro Vesperoni, Bruno Comini, Francesco Castelli, Lina Rachele Tomasoni

**Affiliations:** Division of Infectious and Tropical Diseases, University of Brescia, Brescia, Italy; Medicus Mundi Italia, Brescia, Italy; Division of Infectious and Tropical Diseases, University of Brescia, Brescia, Italy; Medicus Mundi Italia, Brescia, Italy; Department of Experimental Medicine and Public Health, School of Advanced Studies, University of Camerino, Camerino, Italy; Tropical Diseases Unit—ASST Spedali Civili of Brescia, Brescia, Italy; Provincial Health Directorate of Inhambane, Inhambane, Moçambique; Medicus Mundi Italia, Brescia, Italy; Medicus Mundi Italia, Brescia, Italy; Division of Infectious and Tropical Diseases, University of Brescia, Brescia, Italy; Division of Infectious and Tropical Diseases, University of Brescia, Brescia, Italy; Division of Infectious and Tropical Diseases, University of Brescia, Brescia, Italy; Division of Infectious and Tropical Diseases, University of Brescia, Brescia, Italy; Medicus Mundi Italia, Brescia, Italy; Division of Infectious and Tropical Diseases, University of Brescia, Brescia, Italy; Medicus Mundi Italia, Brescia, Italy; Tropical Diseases Unit—ASST Spedali Civili of Brescia, Brescia, Italy

**Keywords:** access to care, ART, HIV, PMTCT, remote areas

## Abstract

**Background:**

Mother-to-child transmission of HIV (MTCT) has, in Mozambique, one of the highest rates (9%). Women living with HIV (WLWH) in remote areas have poor access to health services, including to antiretroviral treatment. On an experimental basis, services to prevent MTCT (PMTCT) are offered in mobile health clinics (*Brigadas Móveis*, BM) in the Inhambane province. The study aims to evaluate the effectiveness of this innovative strategy in the cascade of care for these patients.

**Methods:**

We collected demographic and clinical data on WLWH, as well as HIV-exposed children, reached at least once by BM strategy, over a 24-month period from 2021 to 2023. We performed a statistical analysis to assess adherence to the care pathway and identify risk factors for HIV infection in children.

**Results:**

Two hundred and forty WLWH and 252 children were included in the study. About 40% of the women received a new HIV diagnosis and for 85% of them the BM offered the first PMTCT visit. A beneficial effect on earlier entry into the PMTCT cascade-of-care was observed, with the median gestational age at entry decreasing from 24 weeks in the first year to 20 weeks in the second. Adherence to scheduled visits was lower for women with a recent HIV diagnosis and for women living in communities not reached monthly. A vertical transmission rate of 11% was estimated for exposed infants born from pregnant women.

**Conclusions:**

HIV vertical transmission is still a major challenge in Mozambique, especially for women from remote communities. Decentralization of services is a key strategy for protecting HIV-exposed children. A recent diagnosis and irregular service delivery are significant risk factors for adherence to the PMTCT course.

## Teaser

Innovative and decentralized strategy for pregnant and/or breastfeeding women living with HIV (WLWH) and their exposed children

## BACKGROUND

Despite decades of global efforts, HIV/AIDS remains a major public health challenge, particularly in sub-Saharan Africa, where it disproportionately affects adolescent girls and young women [1]. Mozambique has one of the highest HIV prevalence rates in the region, 12.4% among adults aged 15–49 years, with provincial variations ranging from 7.9% to 20.9%. Prevalence is notably higher among women (15.4%) than men (10.1%) [2, 3]. Moreover, Mozambique's high HIV prevalence exists within a complex health landscape characterized by a heavy burden of co-endemic diseases, including malaria, tuberculosis, and climate-sensitive waterborne illnesses [4, 5].

Among pregnant and breastfeeding women (PW/BW), HIV prevalence is estimated at 9%, posing a significant risk of vertical transmission (VT). National estimates report 140 000 children living with HIV, representing 6% of the global pediatric HIV burden, with around 11 000 new pediatric infections each year [6].

To mitigate this risk, Mozambique's Ministry of Health (*Ministério da Saúde*, MISAU) has developed key strategies: the rollout of prevention of mother-to-children transmission (PMTCT) services in 2004, the adoption of Option B + in 2013 (immediate start and lifelong antiretroviral therapy (ART) for all HIV-positive PW), and a national plan for elimination of VT of HIV, syphilis, and hepatitis B in 2020 [7, 8]. These measures have contributed to progress. However, MISAU's demographic modeling software (Spectrum) suggests that VT has never fallen below 10% nationally, with a rate of 15% in 2018 and a projected 9% for 2024 [6].

A major obstacle remains poor access to health services in remote areas. Nearly 70% of Mozambique's population is rural, and many communities are distant from health facilities. In Inhambane Province, for example, numerous villages require over an hour of travel to reach the nearest health center (HC) [9].

To bridge this gap, since 1979 MISAU launched *Brigadas Móveis* (BM) strategy—mobile health clinics that reach communities over 7 kilometers from the nearest HC, on a monthly or quarterly basis [10]. BM delivers essential services, including maternal and child health (ante e post-natal care), nutrition, immunization, counseling and basic clinical care. In some selected remote communities, reached on a monthly basis, distribution of ART, other than clinical visits and treatment of opportunistic infections, is possible [11–14]. Despite their strategic importance, HIV testing and PMTCT services are not systematically included in BM activities. As a result, a large proportion of WLWH and infants perinatally exposed (IPEs) from most remote communities turn out to be covered by these services.

### Medicus Mundi Italia

Medicus Mundi Italia (MMI) is an international NGO focused on strengthening health systems through community engagement, infrastructure development, and health worker training. In order to enhance service delivery in remote areas of Mozambique, MMI has supported a regular and integrated model of BM, with more than 100 communities reached every year over an area of more than 500 000 inhabitants in Inhambane Province [11, 12]. Thanks to the MMI experience in Inhambane communities and the collaboration with local authorities (*Serviço Distrital de Saúde, Mulher e Acção Social—*SDSMAS, and *Direcção Provincial de Saúde*—DPSI), PMTCT services have been integrated into every BM visit in the 5 supported districts since 2021. In the same year, in collaboration with the University of Brescia, the “*ProTeggiMi”* project was started, with the aim to implement PMTCT services to all the communities reached by the BM, providing ART for HIV-positive PW/BW and ART prophylaxis for IPEs, strengthening adherence to the diagnostic and therapeutic pathway and improving linkage to care, as required by the MISAU protocols. From January 2023, a research project “*REACH”* gave continuity to the community activities of PMTCT. Moreover, some innovative strategies of service decentralization were introduced, including a Point-of-Care equipment (POC or mobile PIMA) that allows the realization of viral load (VL) for PW/BW living with HIV and PCR test as a diagnostic tool for IPEs.

The aim of the current study is to evaluate trends in the uptake of PMTCT decentralized services among WLWH and their infants in the context of the BM operating in rural areas of Inhambane Province, as part of the broader “*ProTeggiMi*” and “*REACH*” initiatives. Additionally, the study sought to identify epidemiological, social, and clinical factors associated with adherence to medical and therapeutic follow-up among mothers and children, in order to address these outcomes in future strategies.

## METHODS

### Study Design

We conducted a prospective, observational study in 4 districts (Massinga, Morrumbene, Homoine and Funhalouro) of the Inhambane province, southern Mozambique. We enrolled pregnant or breastfeeding WLWH (≥14 years old) that accessed to PMTCT services from 1 August 2021 to 31 July 2023. We included women who were visited at least once in BM supported by the *ProTeggiMi* project and included in the PMTCT program, regardless of where they started the follow-up. The frequency of BM visits for each community varied from monthly to quarterly according to a pre-established schedule and remained unchanged throughout the study period [12].

According to MISAU indications, women were tested for HIV using a rapid fourth generation test with high sensitivity (Determine™ HIV-1/2); the diagnosis was confirmed by another rapid test with higher specificity (Uni-Gold™). Conversely, children from the 28th days of life were tested with a qualitative molecular PCR, repeated at 9 months. Seronegativity for HIV was confirmed by a rapid test performed at least 3 months after definitive weaning.

IPEs that were visited at least once by mobile clinics during child health consultations, were recruited and matched with their HIV-infected mothers.

For each patient (woman or child), we calculated an individual adherence percentage. The numerator was the number of visits attended within the patient's specific follow-up period (from first to last visit). The denominator was the total number of visits scheduled in that same period. Attendance at a scheduled visit and/or the collection of an antiretroviral therapy (ART) prescription was used as indirect markers of adherence. For the adherence analysis, we included WLWH and IPEs with a minimum follow-up period of 3 months. This window represents a critical period to observe initial patterns of response to ART and is clinically meaningful, as it aligns with the typical interval between key antenatal visits, allowing for monitoring and intervention before delivery. We classified as non-adherent those WLWH who did not have a follow-up visit or ART dispensing.

In accordance with MISAU definitions, we considered loss to follow-up (LTFU) those WLWH who missed at least 3 consecutive scheduled visits and did not return during the observation period. WLWH referred to HC out from the intervention area were classified as transferred.

For IPEs, outcomes included: HIV-negative, HIV-positive, dead, LFTU and transferred. HIV-negative but still in follow-up in May 2025 were registered.

IPEs diagnosed with HIV were referred to the nearest HC for treatment, as pediatric ART was not yet available within the Mobile Clinics at the time of the study in the majority of the communities.

### Data Management

Data on pregnant and breastfeeding WLWH and their children were collected and stored in Microsoft Excel. Only researchers and the principal research team had access, ensuring data security and confidentiality. We collected data on sociodemographic, clinical and epidemiological factors. Moreover, data on adherence to ART and to medical visits, site of delivery, testing diagnostic procedures, as outcomes of HIV exposed children were collected. VL of WLWH was collected when performed, as in the MISAU protocol: once for every pregnancy or earlier if the load is higher than 1000 cp/mcl, 3 months after delivery. We used record books and clinical tools in use by MMI as per indication of the Ministry of Health as data sources.

### Statistical Analysis

EpiInfo 7.2.4 software has been used for statistical analysis. Descriptive statistics (counts, proportions, means with standard deviation, medians with interquartile range) have been calculated. Comparative analyses included chi-square or Fisher's exact tests for categorical variables and t-tests or ANOVA for numerical variables. Regression models analysed adherence to PMTCT outcomes, incorporating variables significant in bivariate analyses or relevant in the literature.

A multivariate analysis was conducted to assess the association between sociodemographic, clinical, and logistical factors and key outcomes, with the aim of identifying barriers to PMTCT service effectiveness and informing future strategies.

## RESULTS

We enrolled a total of 240 WLWH in the study, who were either pregnant and/or breastfeeding. During the same period, 252 newborns/infants were exposed to the risk of HIV infection through vertical transmission. The number of IPEs exceeds that of WLWH because 4 women had twin pregnancies, 3 experienced 2 separate pregnancies during the study period, 5 infants were born to mothers who were enrolled slightly before or during another pregnancy.

The median age at enrollment was 26 years old (IQR 23–33; Table 1). Educational data were available for 213 women (Table 1). One in 3 patients had no formal education, with this proportion rising to nearly 50% in the Funhalouro district (48%, OR 4.2; *P* < .0001; OR adjusted for age 4.4) Moreover, the prevalence of illiteracy increased with age across the entire cohort (AOR 1.4 per 5 years; *P* = .007; Table 1).

**Table 1.** Characteristic of the Population of Women Living With HIV (WLWH)

	Funhalouro	Massinga	Morrumbene	Homoine	Total
WLWH (n, %)	98 (41%)	69 (29%)	47 (19%)	26 (11%)	240
Pregnant	49 (50%)	46 (67%)	21 (45%)	13 (50%)	129 (53%)
Breastfeeding	49 (50%)	23 (33%)	26 (55%)	13 (50%)	111 (47%)
Median age (y, range)	27	26	26	26	26
Schooling (n = 213) (n, %)					
None	44 (48%)	13 (18.8%)	9 (23.7%)	1 (3.7%)	64 (30%)
5 y	38 (42%)	27 (39.1%)	18 (47.4%)	18 (66.7%)	92 (43%)
7 y	1 (1.1%)	3 (4.3%)	6 (15.8%)	4 (14.8%)	14 (6.6%)
10 y	8 (8.8%)	23 (33.3%)	5 (13.2%)	4 (14.8%)	40 (19%)
> 10 y	0	3 (4.3%)	0	0	3 (1.4%)
Site of enrollment in PMTCT services (n, %)					
*Brigada Móvel*	67 (68%)	68 (99%)	44 (94%)	28 (97%)	185 (7%)
Health Center	31 (32%)	1 (1%)	3 (6%)	1 (3%)	55 (23%)
First HIV diagnosis during pregnancy/breastfeeding (n, %)					
* Brigada Móvel*	23 (74%)	33 (87%)	15 (88%)	9 (82%)	81 (84%)
Health Center	8 (16%)	5 (13%)	2 (12%)	2 (18%)	16 (16%)
Site of delivery (n = 190) (n, %)					
Home	46 (54.8%)	40 (80%)	12 (37.5%)	11 (45.8%)	109 (7.4%)
Health Center	38 (45.2%)	10 (20%)	20 (62.5%)	13 (54.2%)	81 (42.6%)

PMTCT service enrollment among WLWH was achieved through BM visits for the majority of participants (77%, n = 185/240), while a minority (23%, n = 55/240) were enrolled at a health center and subsequently followed up through the BM service. Enrollment via BM was significantly more likely in the Massinga and Morrumbene districts compared with Funhalouro (OR 3.3 and 8.2, respectively; *P* = .003 and .0008, respectively). Enrollment through BM was less likely when BM sessions were not held monthly (24/38 days vs 165/206 days; OR 0.4; 0.2–0.9; *P* = .03). Additionally, even after adjusting for the above associations, the likelihood of enrollment in BM doubled with each additional semester of project implementation (AOR 2.1; 1.5–3; *P* = .0001).

On average, 85% of visits were conducted in BM (SD 0.2), whereas 122 WLWH (58.4%, 95% CI 51.4–65.1%) were followed exclusively in the context of BM without attending any HC during the entire follow-up period. In the Funhalouro district, however, the patients’ management was mixed between HC and BM for 52/91 women (57%) (*P* < .0001).

**Graph 1.**
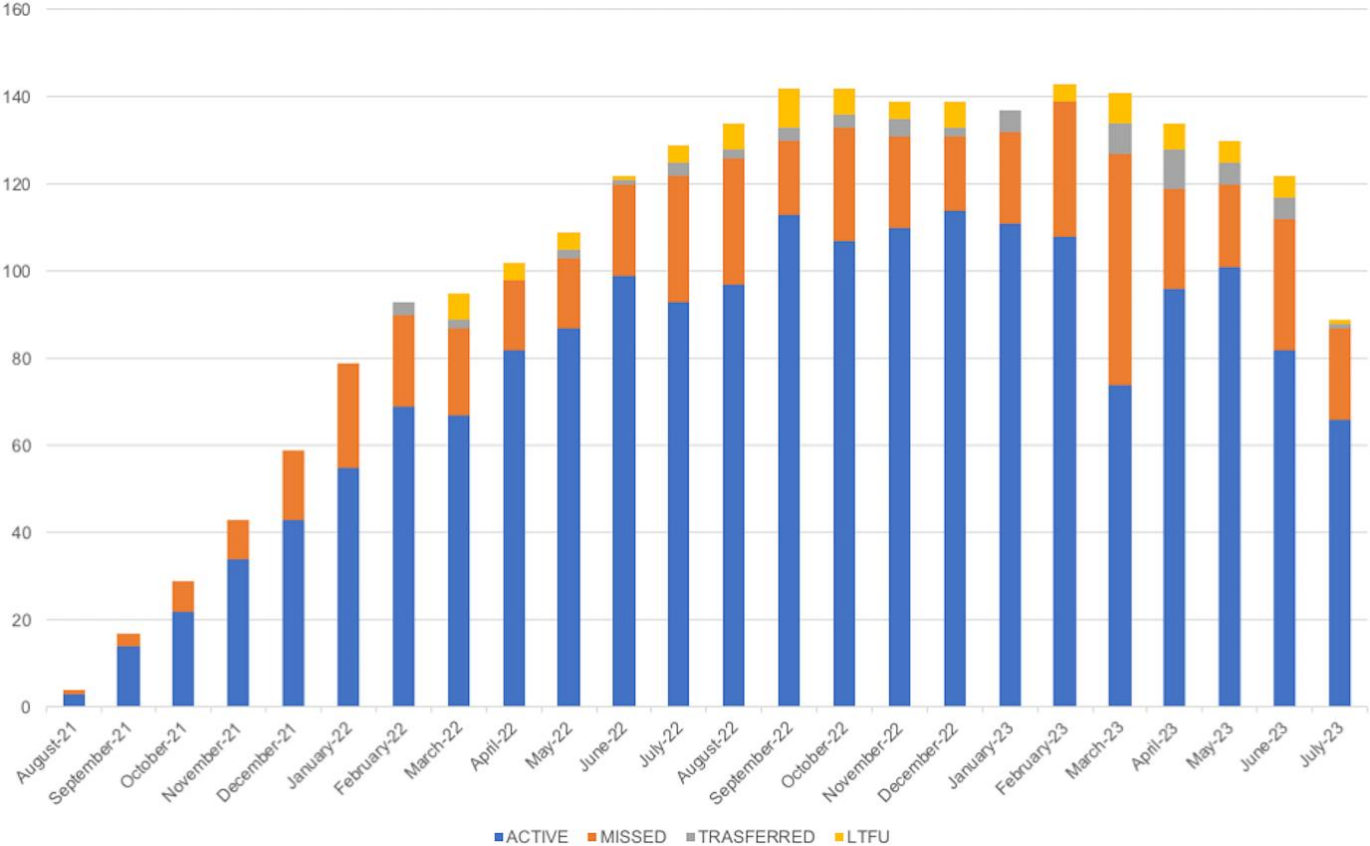
Trend of cumulative adherence to scheduled appointments in the WLWH population in follow-up in the PMTCT services during the study period.

In the overall population, the median duration of follow-up was 9 months (IQR 5–14), without significant difference between districts. Adherence was calculated only among women with an observation period of at least 3 months (n = 210) and it was 83% (median; IQR 66–95). Adherence was significantly and independently lower among WLWH with a very recent HIV diagnosis (median 74%, IQR 62–87; *P* = .0002), and even lower for WLWH living in communities not served by monthly BM visits (median 72%, IQR 60–87; *P* = .001) (Graph 1).

For the 40% of the women (96/240), entry into PMTCT services coincided with their HIV diagnosis, and 83.5% of these diagnoses occurred during BM visit. Among these, 64% (61/96) were pregnant WLWH and 36% (35/96) were breastfeeding. The likelihood of identifying new HIV diagnoses among pregnant/breastfeeding WLWH was significantly higher in the Massinga district (87%, OR 2.3; 1.3–4.1, *P* = .003), and significantly lower in Funhalouro (74%, OR 0.5; 0.3–0.9, *P* = .02). Moreover, of the 185 women who entered PMTCT services for the first time in the BM, the 43% (n = 80) had no previous HIV diagnosis, compared to 29% (16/55) of those who entered PMTCT at the HC (OR 1.8; 0.94–3.4, *P* = .04; one-tailed mid-*P* exact test). Among those with a previous HIV diagnosis (n = 144), 16% of WLWH (23/144) received their diagnosis between 2010 and 2015, 52% (75/144) between 2016 and 2020% and 32% (46/144) between 2021 and 2022.

### Outcomes in the WLWH Population Linked to Care During Pregnancy

One hundred twenty nine WLWH had 135 pregnancies events, as 3 women were followed-up for 2 consecutive pregnancies and 3 had twin pregnancies. The median duration of antenatal follow-up was 4 months (IQR 3–6). On average, enrollment in PMTCT occurred 3.5 months before delivery (IQR 2–5). The timing of enrollment improved over the course of the project: by the third semester of activities, enrollment occurred approximately one month earlier (4.4 months, SD 2.4, vs 3.4 months, SD 2) (*P* = .04) (Graphic 2). Only 15.7% of pregnant WLWH attended all 3 visits scheduled in trimester before delivery. The median number of visits during this phase of pregnancy was 2, but it was significantly higher for women who gave birth in a HC (2.5, SD 1.1) compared to those who gave birth at home (1.9, SD 1.2, *P* = .03).

**Graph 2.**
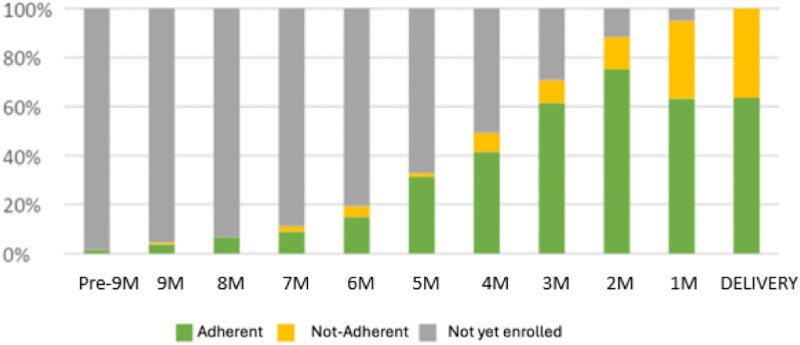
Time of enrollment during pregnancy and adherence to follow-up visits of pregnant WLWH.

VL was required for 45/110 eligible pregnant WLWH (41%), with 20 tested within the interval of 8 weeks before to 1 week after delivery. Among those who received the test results, 74% (28/38) had a VL inferior to 1000 copies/ml, while the 26% had a median VL of 7353 copies/ml (IQR 3930–28 538 copies/ml).

**Figure 1.**
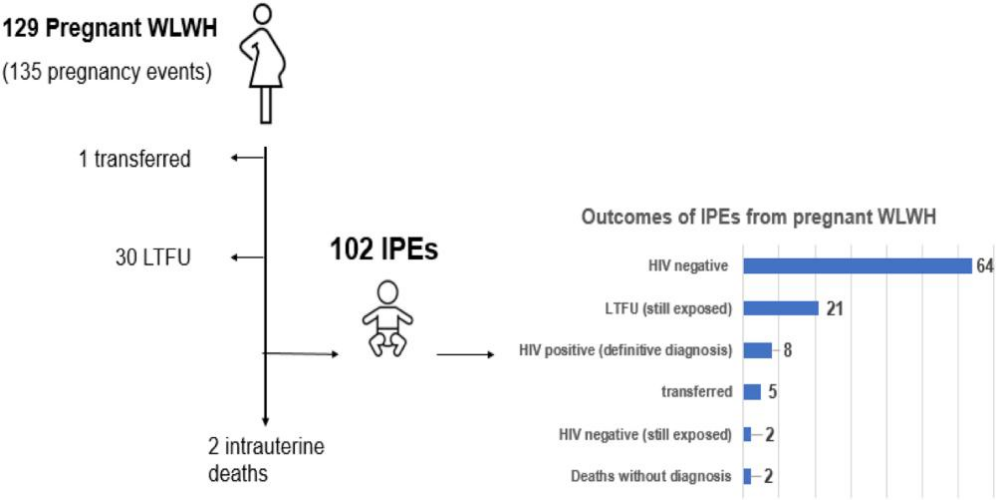
Outcome of the pregnant WLWH and of their newborns by May 2025.

Therefore, 135 potential infants exposed to vertical transmission were initially estimated from 129 PW and outcomes are explained in Figure 1. Loss to follow-up was more likely among pregnant WLWH in communities served by bi-monthly BM [4/16 versus 10/119, OR 3.6, 0.98–13.4 (*P* = .038; one-tailed exact Mid-*P*)], any significant difference was registered among districts. Outcomes of 102 out of 135 IPEs, whose data were available, were evaluated by May 2025. Eighty one newborns (74.3%, 95% CI 65.4–81.6) were enrolled within the first 2 months of life, while others later. For 88.9% of these newborns (72/81, 95% CI 80–94), the PCR testing was performed at the first visit, as suggested by MISAU. Among children that tested positive to HIV, the 50% (4/8) was already positive at the first PCR performed before 2 months of life. The estimated vertical transmission rate, calculated considering the HIV-positive children among IPEs with definitive diagnosis (either seropositive or seronegative), was 11% (8/72, 95% CI 4.9–20.7), half of which is determined by perinatal transmission (4/72, 5.5%). Moreover, VT rate was significantly higher for IPEs born to mothers diagnosed with HIV at ante natal care entry (6/26, 23% vs 2/46 4,3%; OR 6.6, IC95% 1.2–35) (*P* = .022). In multivariate analysis, the likelihood of loss to follow-up was significantly higher among IPEs born to WLWH who received their first diagnosis of HIV during pregnancy (AOR 9.8; 95% CI: 1.1–88, *P* = .04), and among those residing in communities served by BM with less-than-monthly visits (AOR 58; 95% CI: 5.6–608, *P* = .0007). Moreover, LTFU was higher in those IPEs whose mothers were not adherent to their pre- and postnatal visits. Maternal adherence among IPEs lost to follow-up was 64% (IQR 44–73), compared to 87% (IQR 71–95) among those who remained in follow-up through the end of the study (*P* = .0004).

### Outcomes in the WLWH Population Linked to Care During Breastfeeding

Among the 240 WLWH in the cohort, 111 women were enrolled in the project after giving birth, thus with a risk of VT due to breastfeeding. For 31.5% of them (35/111; 95% CI: 23–40) the enrollment coincided with their first HIV diagnosis. In 79% of these cases, enrollment occurred in BM. Entry into PTMTC services occurred at a median of 8 months postpartum (IQR: 4.4–11.6). In terms of adherence, 82% of BW (median value; IQR: 64–93) were adherent to scheduled visits, and 88% of these took place in BM. VL testing was performed in 17% (19/111) of breastfeeding WLWH, of whom 62.5% (10/16) had a VL inferior to 1000 cp/ml, since 3 test results were missing.

**Figure 2.**
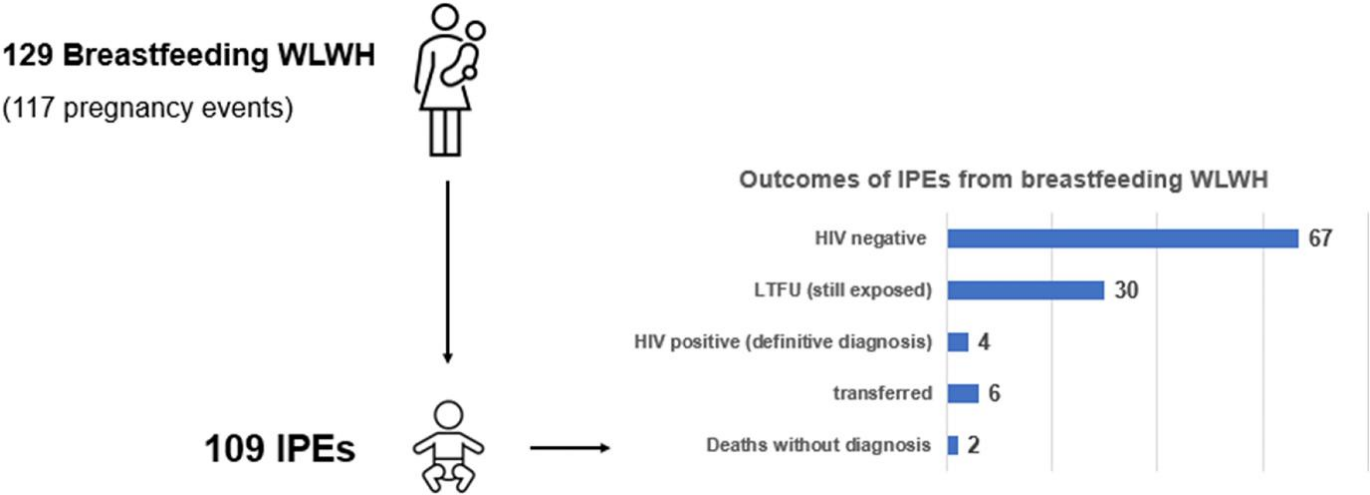
Outcomes of breastfeeding WLWH and their IPEs by May 2025.

The outcomes of their 113 children (including one set of twins and one woman with 2 children) were known for 109 at the end of the project, as shown in Figure 2. About 84/105 underwent PCR testing (80%; 95% CI: 71–86): 81% at the time of PTV enrollment and 81% (22 among the 27 eligible) within 2 months of life. Among the 4 IPEs that tested positive for HIV, 3 started ART and were alive by the end of the study, while one child died.

In a similar way to what was observed in the pregnant WLWH population, a higher rate of early dropout from the PMTCT pathway was present in those communities where BM visits were programmed less than once per month (52.6% vs 18%, *P* = .002).

Considering the global IPEs population with available data (n = 211 children), we studied the median age at the time of attainment of the outcome: the median age for LTFU (n = 51) was 13 months (IQR 9–17), 20 months for negative (n = 131, IQR 18.5–21) and 9 months for HIV positive children (n = 12, IQR 2.6–11.6)

## DISCUSSION

This study sheds light on interventions to PTMCT of HIV in a large cohort of mothers and children with limited access to health care. In our cohort, the proportion of women newly diagnosed with HIV during pregnancy was slightly higher than national data, where 29.3% of women living with HIV (WLWH) initiate ART during pregnancy [2]. This may be partially explained by the mobile clinic (BM) strategy, which reaches women with limited health access and facilitates earlier entry into PMTCT services (mean gestational age at enrollment improved from 3.5 to 4.4 months, *P* = .04). It remains unclear whether these WLWH experienced more barriers to HIV testing before pregnancy or whether they were already aware of their status. Despite routine cross-verification of first diagnoses by local health workers, this process is complex. As highlighted in other studies [15–17], WLWH may undergo multiple HIV tests due to denial, stigma, and social isolation, which hinder acceptance of the diagnosis. Additionally, the substantial number of women diagnosed during breastfeeding raises concern that a notable proportion of pregnant women may bypass the opt-out HIV testing strategy during antenatal care.

Adherence to scheduled visits among pregnant WLWH was especially poor in the third trimester (15.7%), a critical period for vertical transmission (VT). This may be due to reduced mobility in late pregnancy or increased family monitoring related to cultural aspects. Notably, WLWH with a recent diagnosis showed lower visit adherence (74%, *P* = .0002), particularly in communities served by mobile clinics less than once a month (72%, *P* = .001). These results are difficult to compare across studies due to varying definitions of adherence [18].

A recent HIV diagnosis, followed by rapid ART initiation, was also associated with higher loss to follow-up (LTFU), consistent with prior evidence [19–21]. Less-than-monthly mobile clinic outreach was linked with higher LTFU rates, suggesting that increasing visit frequency may improve service uptake and retention, especially in in areas with limited healthcare access.

Among pregnant women eligible for VL testing during the peripartum period, only 41% (45/110) were tested, and of these, 74% had a VL <1000 copies/mL. This limited coverage was consistent with national guidelines at the time, which did not require routine VL testing at delivery. Including breastfeeding WLWH (n = 19), virological suppression in our cohort was 77% (64/83), comparable to national rates: 72% in 2018, 61% in 2021, and 74% in a rural cohort from the same province [2, 3, 22]. Median VL during the peripartum period was 7.353 copies/mL, a level previously associated with VT rates between 5.3% and 14.7% [23]. Although reasons for detectable VL were not explored in this study, this represents a critical area for future investigation, particularly in light of Mozambique's documented burden of drug resistance [24].

Overall LTFU was 20.6% among pregnant women and 25% among breastfed HIV-exposed infants. LTFU was more frequent among IPEs whose mothers received care from less frequent mobile outreach (52.6% vs 18%). Recent maternal diagnosis was also associated with infant drop-out from care. Poor retention among PW/BW and their infants remains a major issue in Mozambique, as in other African contexts. Contributing factors include stigma, lack of social support, long wait times, crowded clinics, delays, and concerns over privacy during counseling [3, 15–19, 25–30].

Regarding early infant diagnosis, 74.3% of IPEs received PCR testing within the first 2 months of life. Among those enrolled in the PMTCT program within this window, 88.9% were tested. For infants of WLWH diagnosed during breastfeeding, 81% underwent PCR testing at program entry, with 81% of eligible infants (22/27) tested within the recommended timeframe. These rates are consistent with national figures (73% in 2018% and 76% in 2021) [3, 31] but remain below the UNAIDS target of 95% [32], underscoring the need for stronger outreach in underserved areas. The VT observed in our cohort was 11.1%, with half of the cases attributed to perinatal transmission. This estimate includes only IPEs born to pregnant WLWH who had a confirmed diagnostic outcome (either seropositive or seronegative). Women enrolled postpartum were excluded from this analysis to avoid survival bias, as some children may have died prior to enrollments (median age of enrollment: 8 months). Overall, using the same calculation methodology, 12/143 infants with a known diagnostic outcome were HIV-positive (8.4%, data shown in Supplementary table 1). These partial outcomes represent a major limitation of the study, impeding comparisons with other cohorts and alignment with national benchmarks. Moreover, missing outcome data for some IPEs could bias the VT rate estimate toward underestimation. Nonetheless, this estimated transmission rate aligns with findings from Inhambane province [6] and others community-based studies [33, 34], which is notable given that this population was accessed for the first time through a mobile outreach strategy. The lack of data on maternal co-infections or other health indicators that could affect PMTCT outcomes, mainly due to absent routine screening and report within the Mozambican public healthcare system, should be also considered.

In addition to these limitations, the study's predominantly quantitative approach could be strengthened by incorporating qualitative methodologies, such as interviews or focus groups, to explore the social and structural barriers affecting PMTCT services, that in similar contexts have provided added value [35–37].

## CONCLUSIONS

This study highlights the ongoing challenges faced by WLWH and children exposed to vertical HIV transmission and emphasized the pivotal role of mobile clinics in providing PMTCT services in rural areas of Mozambique, that provide HIV services while addressing logistical, social, and cultural barriers to care. The specific subpopulation of women reached by this decentralized strategy may not be representative of the general population in this context either (eg,: socio-cultural aspects, familiarity with mobile health services, clinical factors). Moreover, there are no comparative data on women not reached by the BM (eg,: women living in the same communities who do not attend the BM service). Evidences in this area suggest that future interventions should prioritize women with recent HIV diagnoses and expand the availability of early infant HIV PCR testing to improve timely diagnosis, retention in care and treatment adherence; increasing the frequency of mobile clinic visits may also strengthen these efforts. By openly acknowledging and discussing this inherent limitation of the mobile clinic model, we strengthen the validity and applicability of our conclusions for programmers and policymakers working in similar low-access settings. The observed declined in adherence during the third trimester of pregnancy and the high rate of non-institutional deliveries underscore the need for tailored public health interventions within this context. Enhancing HIV counselling and support for ART initiation, alongside with peripartum VL monitoring and adherence reinforcement, are essential to reduce vertical transmission rates. Additionally, the implementation of HIV drug resistance testing for individuals with detectable VL, currently unavailable in Mozambique's national HIV program, could significantly improve clinical management and outcomes. Finally, the BM model's principal strength lies in its adaptability to both local needs and the specific characteristics of the Mozambican health system, making it a viable strategy for mitigating healthcare access disparities. Its successful replication in other contexts, however, depends on critical factors such as seamless integration with existing health centers, the availability of trained personnel, and demonstrable cost-efficiency.

## Supplementary Material

ofaf647_Supplementary_Data

## Supplementary Data

Supplementary materials are available at *Open Forum Infectious Diseases* online. Consisting of data provided by the authors to benefit the reader, the posted materials are not copyedited and are the sole responsibility of the authors, so questions or comments should be addressed to the corresponding author.
