# Supplementary figures and images for: “ProTeggiMI”: Adherence to HIV Vertical Transmission Prevention Pathway in Remote Communities in Mozambique
Source: Open Forum Infect Dis. 2025 Oct 15;12(11):ofaf647. doi: 10.1093/ofid/ofaf647 (PMC12582313; doi:10.1093/ofid/ofaf647)

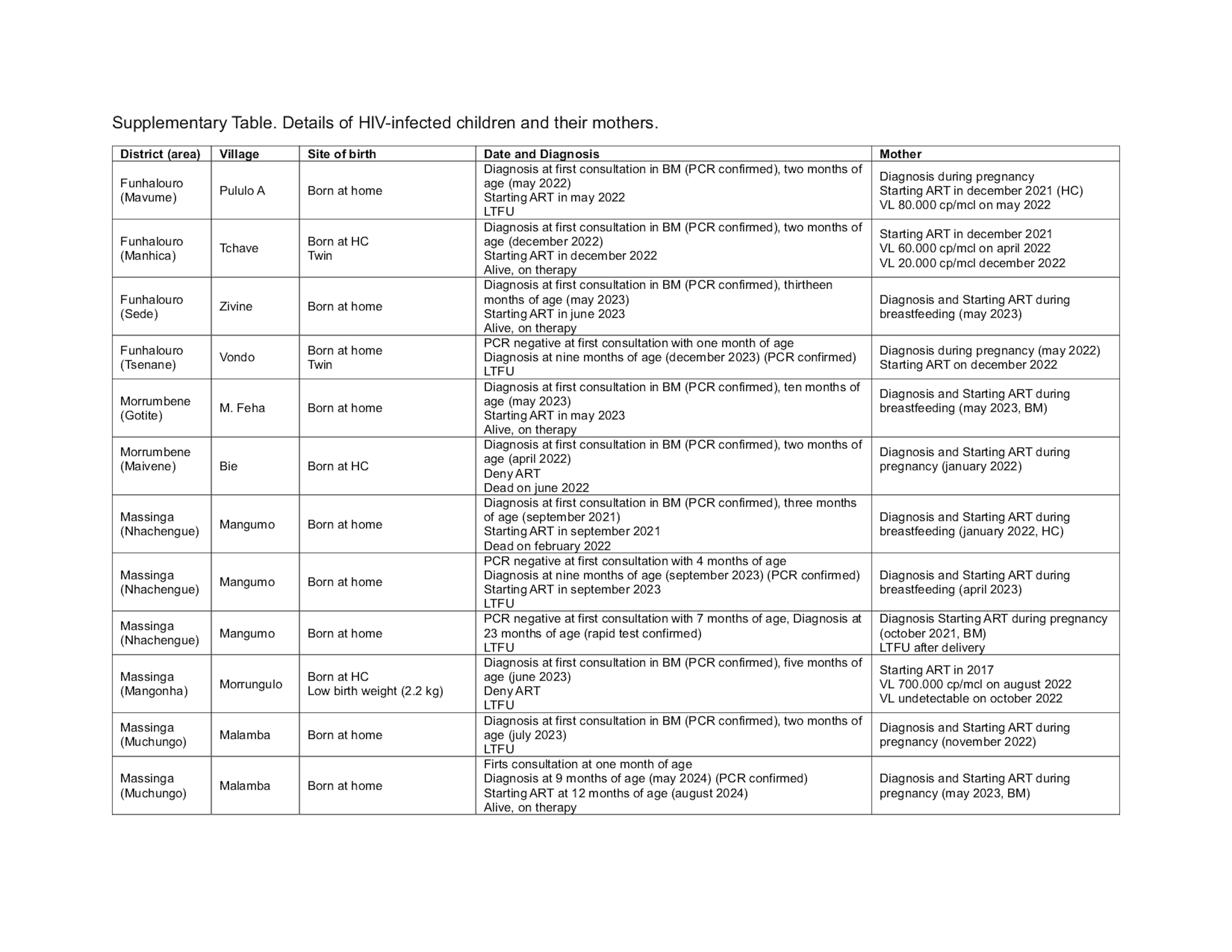

Supplement: ofaf647_Supplementary_Data [file ofaf647_supplementary_data.jpeg]
